# An efficient Co_3_S_4_/CoP hybrid catalyst for electrocatalytic hydrogen evolution

**DOI:** 10.1038/s41598-017-12332-4

**Published:** 2017-09-19

**Authors:** Tingting Wang, Liqian Wu, Xiaobing Xu, Yuan Sun, Yuanqi Wang, Wei Zhong, Youwei Du

**Affiliations:** 1 Collaborative Innovation Center of Advanced Microstructures, National Laboratory of Solid State Microstructures and Jiangsu Provincial Laboratory for NanoTechnology, Nanjing University, Nanjing, 210093 China; 2 College of electronic Engineering, Nanjing Xiaozhuang University, Nanjing, 210017 China

## Abstract

The development of efficient, universal and inexpensive electrocatalysts for hydrogen evolution reaction (HER) is central to the area of sustainable energy conversion. Considering the Co-based sulfides/phosphides have the same catalytic mechanism with the hydrogenases occurring in nature. Here, a new catalyst based on Co_3_S_4_/CoP hybrid that is comprised entirely cheap and earthabundant elements, was first synthesized via a two-step method, the Co(CO_3_)_0.5_(OH)·0.11H_2_O precursor was prepared by a hydrothermal method, followed by phosphidation and sulphidation under Ar atmosphere simultaneously. The resulting Co_3_S_4_/CoP hybrid material possessed porous core-shell structure with a homogeneous element distribution and large electroactive surface area (~21.04 mF cm^−2^). More importantly, the nanostructured Co_3_S_4_/CoP electrode exhibits excellent HER properties in acid medium with a low onset overpotential of 34 mV, a small Tafel slope of 45 mV dec^−1^, as well as a large exchange current density of 150 μA cm^−2^. These results obtained in this study indicate that the Co_3_S_4_/CoP hybrid nanorod is promising replacement to the Pt-based catalysts for H_2_ production. Moreover, the synthetic method presented in this work can provide an efficient way to synthesis other nanocomposites.

## Introduction

The limited reserves of carbon-based fuels (e.g. oil, ethanol and coal) and the environmental hazards associated with the use of these fuels have triggered the research community to find an abundant, zero-emitting and eco-friendly energy source as an alternate energy server very urgently^1–3^. Hydrogen (H_2_), as a clean, convenient and pollution-free energy source has aroused substantial research interest^4–7^. Of varied methods, water electrolysis driven by renewable-resource-derived electricity is regarded as an ideal pathway for hydrogen production^8^. However, due to the sluggish kinetics, the high-performance electrocatalyst and the overpotential have to be applied to overcome the energy barriers inherent to hydrogen evolution reaction (HER)^9,10^. At present, Pt-based noble metal materials remain the most efficient electrocatalysts for HER under acidic medium, but the high cost and the scarcity limit their large-scale applications^11,12^. Therefore, it is urgent to develop non-noble metal or metal-free catalysts for HER. Considering the hybrid catalyst are more likely to achieve their potentials in physical, chemical, and electronic properties than the catalyst with a single component or configuration^13^, various hybrid catalysts have been synthesized and investigated. For example, Li *et al*.^14^ synthesized MoS_2_/RGO hybrid with a high HER performance by establishing a strong chemical and electronic coupling between MoS_2_ and GO; Pan *et al*.^15^ investigated the HER performance of CoP/MoS_2_-CNTs hybrid, and attributed the remarkable catalytic activity to the strong synergistic effect between CoP and MoS_2_, and the excellent electrical conductivity of CNTs. Similar promoting effects have also been demonstrated on Ni_2_P-G/NF^16^, MoS_2_/Fe_3_O_4_^17^ and Co_9_S_8_-MoS_x_^18^ hybrid catalysts for hydrogen evolution.

Co-based sulfides/phosphides have been widely used in classical hydrodesulfurization (HDS) reaction, which has the similar requirements for binding energy of hydrogen on surfaces with HER reaction^19–21^. Moreover, previous studies have also reported that the catalytic mechanism of Co-based sulfides/phosphides is the same as the hydrogenases occurring in nature^22^. Therefore, it is expected that Co-based sulfides/phosphides can also be used as efficient catalysts for HER. There are five intermediate phases (Co_4_S_3±y_, Co_9_S_8_, Co_1−y_S, Co_3_S_4_ and CoS_2_) in the binary Co-S phase diagram^23^. Similarly, cobalt phosphides also have a serious of compositions^24^, such as Co_2_P, CoP, and CoP_2_. Among these phases, both Co_3_S_4_ and CoP are considered as the competitive candidate for utilization in HER, because the attractive metallic nature of Co_3_S_4_ and the moderate ΔG_H*_ of CoP can ensure the fast charge migration^25–27^ and the effective adsorption/desorption during electrocatalytic process, respectively. However, to the best of our knowledge, the synergistic effect argument for the Co_3_S_4_/CoP hybrid is yet to be investigated for HER reaction.

In this context, we have successfully synthesized porous Co_3_S_4_/CoP nanorods (NRs) via a facile two-step synthetic strategy. As expected, the as-prepared Co_3_S_4_/CoP catalyst exhibited an excellent HER catalytic activity and good long-term stability in acidic medium. Moreover, pure Co_3_S_4_ and CoP catalysts were also synthesized and their electrocatalytic performances for the HER were measured for comparison. The results indicated that the synergistic effect between Co_3_S_4_ and CoP plays an important role in enhancing the HER performance, and the possible mechanisms accounting for these results are discussed.

## Results

### Characterization of morphology and structure

**Figure 1:**
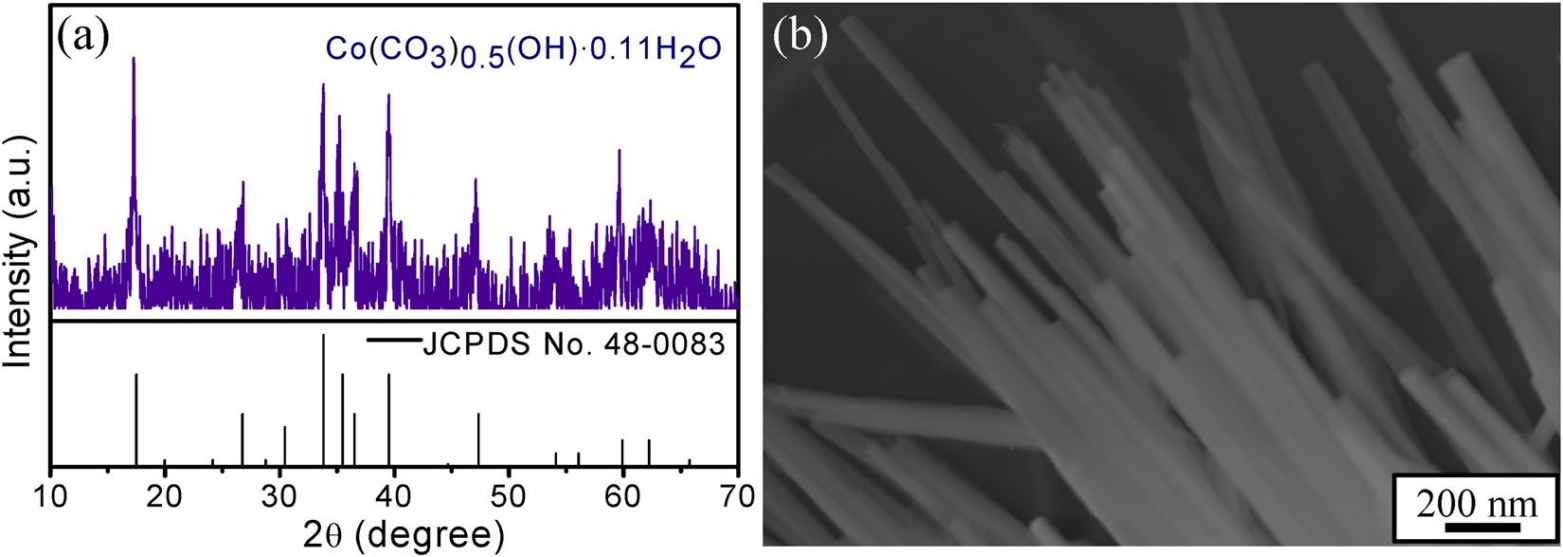
(**a**) Powder XRD spectrum, (**b**) SEM image of the cobalt precursor.

Firstly, the structure and morphology of cobalt precursor were investigated and shown in Fig. 1. Diffraction peaks appearing in the precursor can be well ascribed to Co(CO_3_)_0.5_(OH)·0.11H_2_O (JCPDS No. 48–0083), and the SEM result indicates that the precursor is composed of a large number of 1D nanorods (average diameter ~68 nm) with smooth surface.

**Figure 2:**
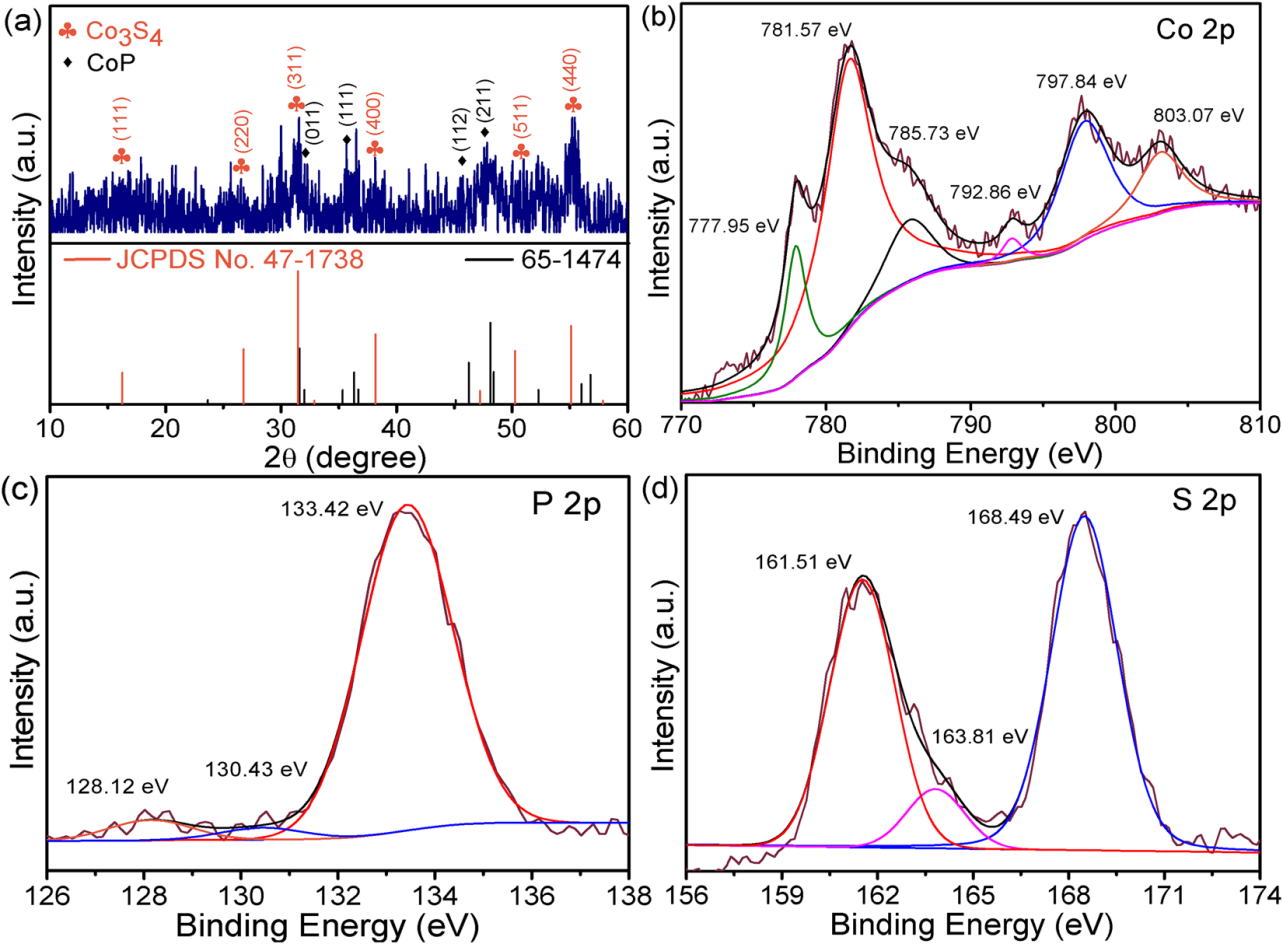
(**a**) XRD pattern and (**b–d**) XPS spectra of the sample.

After phosphidation and sulphidation, the final product was characterized by XRD to ascertain the crystal phase. As depicted in Fig. 2(a), the diffraction peaks located at 16.7°, 26.5°, 31.3°, 38.1°, 50.7° and 55.2° can be well indexed, respectively, to (111), (220), (311), (400), (511) and (440) planes of Co_3_S_4_ (JCPDS No. 47–1738 cell parameters: *a* = *b* = *c* = 0.942 nm). The peaks located at 32.1°, 35.8°, 45.7°, 38.1° and 48.1° correspond to the (011), (111), (112) and (211) planes of CoP (JCPDS No. 65–1474, cell parameters: *a* = 0.507 nm, *b* = 0.328 nm, *c* = 0.558 nm). In addition, a peak at 30.1° can also be observed, which is attributed to the deposition of excess sulfur. All these results indicate that the sample is a hybrid material compounded by Co_3_S_4_ and CoP, and the width of the peaks broadens obviously, implying weak crystallization of the sample. The EDX analysis (Supplementary Fig. S3) is further confirming the composition of the product.

Subsequently, the detailed valence states of the Co_3_S_4_/CoP hybrid catalyst were studied by XPS (Fig. 2(b–d)). For the Co 2p core level, two regions are apparent: one region is belong to Co 2p_3/2_, including two peaks at 777.95 and 781.57, along with one satellite peak at 785.73 eV; the other is belong to Co 2p_1/2_, two peaks at 792.86, 797.84, being accompanied by a satellite peak at 803.07 eV are included as well, suggesting the coexistence of Co^2+^ and Co^3+^ in the sample^28^. In the P 2p spectrum, the peaks with binding energies of 128.12 and 130.43 eV can be attributed to P 2p_3/2_ and P 2p_1/2_ from CoP, respectively, while the one at 133.42 eV corresponds to P-O^29^. For the case of S, the asymmetric peak observed at lower binding energy can be fitted into two peaks at 161.51 and 163.81 eV correspond to S 2p_3/2_ and S 2p_1/2_ states in Co_3_S_4_^30^, and the symmetric peak observed at higher binding energy of 168.49 eV can be assigned to oxidized S species due to air contact.

**Figure 3:**
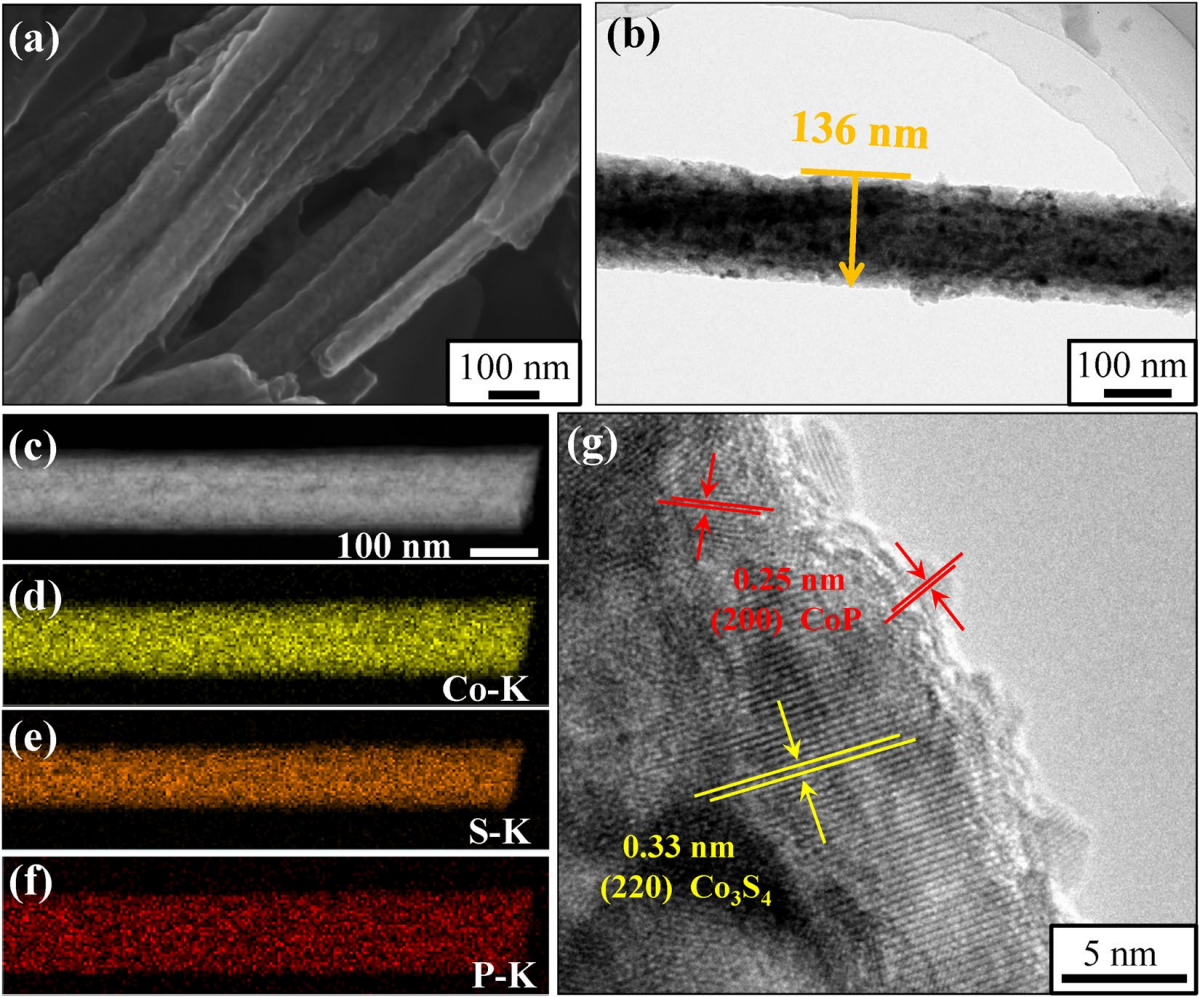
**(a**) SEM, (**b**) TEM, (**c–f**) EDS mapping and (**g**) HRTEM images of Co_3_S_4_/CoP NRs.

SEM image shown in Fig. 3(a) demonstrates that the rod-like morphology of the Co_3_S_4_/CoP is completely inherited from the Co(CO_3_)_0.5_(OH)·0.11H_2_O precursor during the annealing process under Ar atmosphere. Moreover, it is noteworthy that, compared with the precursor, both the surface roughness and average diameter (~140 nm) of Co_3_S_4_/CoP NRs are significantly enlarged. Such morphology would be an ideal structure for HER, because the rough surface of hybrid guarantees a high active-site density, which is beneficial providing efficient catalytic activity for surface electrochemical reactions, and the one-dimensional (1D) nanostructure can also provide channels and few crystal boundaries for fast charge transport pathways with reduced scattering^31^. Figure 3(b) displays the TEM image of a part of single Co_3_S_4_/CoP nanorod under high magnification, light and dark areas within this image further confirm the surface of Co_3_S_4_/CoP NRs is concave–convex. In order to investigate the distribution of elements, the corresponding EDS mappings of elemental Co, S and P from the TEM image of Fig. 3(c) are carried out. It is evident from Fig. 3(d–f) that the distribution of these three elements is homogeneous, and the distribution of elemental P is slightly wider than that of S, suggesting the hybrid is a core-shell structure with the CoP as the shell (thickness ranging from 2–10 nm) and Co_3_S_4_ as the core. Additionally, the HRTEM (Fig. 3(g)) image is given to explore the microstructure of Co_3_S_4_/CoP hybrid in detail, the fringe spacing of 0.33 and 0.25 nm is observed, corresponding to the (220) plane of Co_3_S_4_ and (200) plane of CoP, respectively. The result is well matched with the XRD analysis.

**Figure 4:**
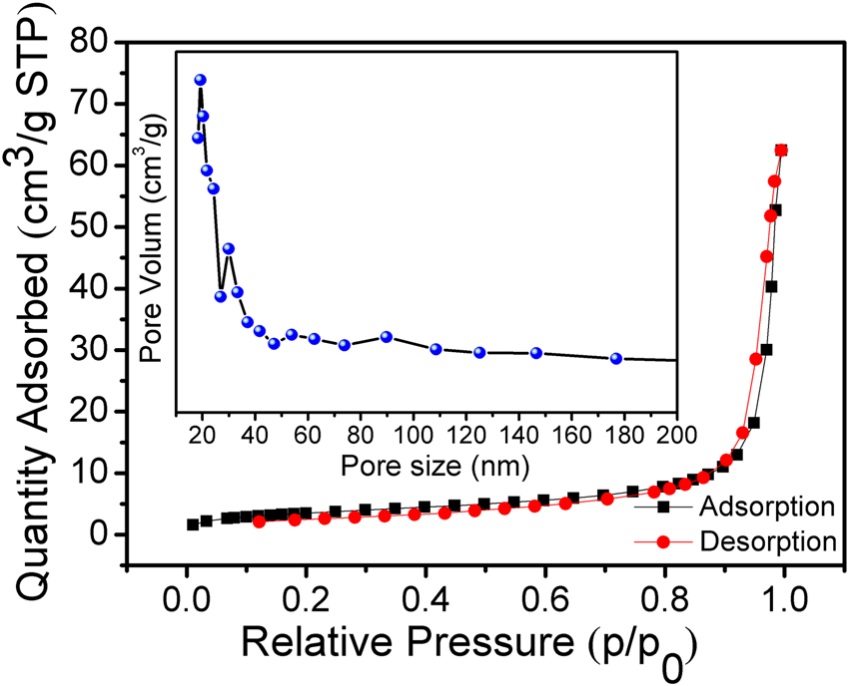
N_2_ adsorption-desorption isotherm and BJH pore size distribution plot (inset) of Co_3_S_4_/CoP hybrid NRs.

Finally, BET measurement is carried out at 77 K to further elucidate the intrinsic property of the sample. As shown in Fig. 4, the N_2_ adsorption/desorption isotherm shows a typical type- IV isotherm with a hysteresisloop above P/P_0_ = 0.9, demonstrating the mesoporous characteristic of the Co_3_S_4_/CoP hybrid NRs. The inset is the corresponding pore size distribution plot, which is calculated from the adsorption branch of nitrogenisotherm and the BJH method. The result indicates that the hybrid contains a number of pores, and most of the pores fall into the size range of 20–25 nm. These pores are attributed to the interparticle spaces, which presumably arise from the gas release during the pyrolysis of the precursor^32^. Compared to solid materials, porous nanostructures can not only possess more active sites, but also allow easy electrolyte infiltration into the inside of the catalysts^33,34^. Moreover, both the porous and 1D structure can promote the formation and release of bubbles from the catalyst/electrode surface, and thus improve the efficiency of energy conversion and reinforce the electrolyte contact with the catalyst.

**Figure 5:**
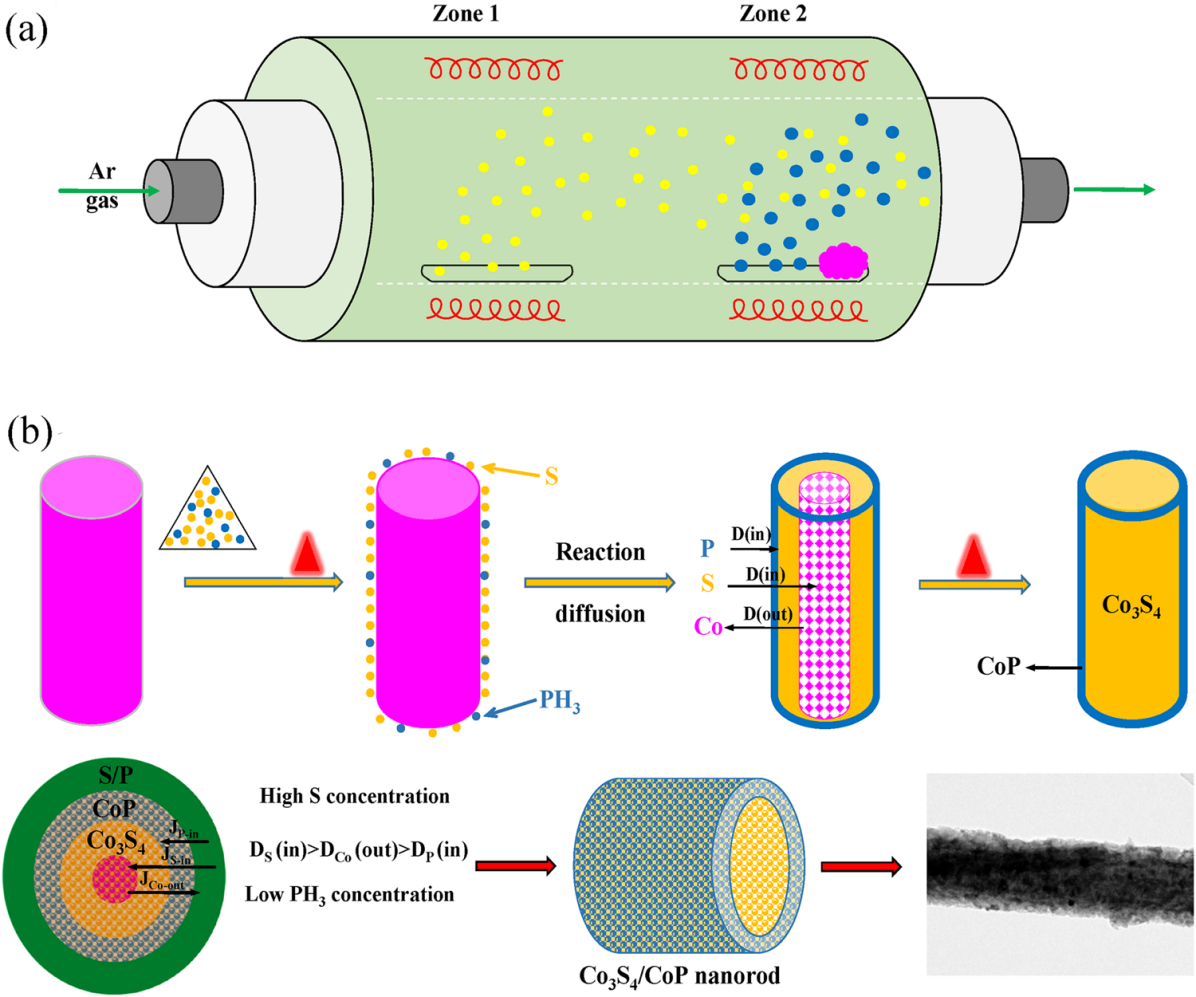
Schematic illustration for the synthesis of Co_3_S_4_/CoP hybrid NRs. (**a**) Schematic diagram of experimental device, (**b**) the formation mechanism of the core-shell structure via the Kirkendall effect.

On the basis of the results above we concluded that, as the template, cobalt precursor creates a strong confinement effect on the synthesis of Co_3_S_4_/CoP NRs, and the formation of core-shell structure is controlled by the Kirkendall effect^35^. As illustrated in Fig. 5(b), when the annealing temperature of two heating zones raise up to the gasification and decomposition temperatures of S and NaH_2_PO_2_, respectively, S vapor and newly generated PH_3_ can be reached and absorbed on the surface of cobalt precursor with the flow of Ar. Then, Co tends to diffuse outward to react with S and P, while S and P have a tendency to diffuse inward driven by the chemical potential and concentration gradient^36^. It is noteworthy that the concentration of S, P and Co sources are different (Co:P:S sources = 1:5:15), and according to Fick’s first law, the diffusion rate of S will be much faster than P and Co. Therefore, the Co_3_S_4_/CoP hybrid with core-shell structure is obtained. Moreover, because of the existence of pores and the bigger atomic radius of S/P compared to O, the mean diameter of final product is much larger than that of precursor.

### Electrocatalytic performance for HER

The electrocatalytic performance of the Co_3_S_4_/CoP catalyst for HER in 0.5 M H_2_SO_4_ solution was systematically evaluated using a standard three-electrode system with an ion-exchange membrane to prevent the redeposition of Pt onto the HER catalyst at room temperature^37,38^, and all the measurements were carried out after purging with high-purity N_2_ for 30 min to get rid of the dissolved oxygen. Moreover, in order to achieve a steady-state condition, more than 20 cycles of cycle voltammetry (CV) tests with scan rate 0.1 V s^−1^ were repeated before data collection.

**Figure 6:**
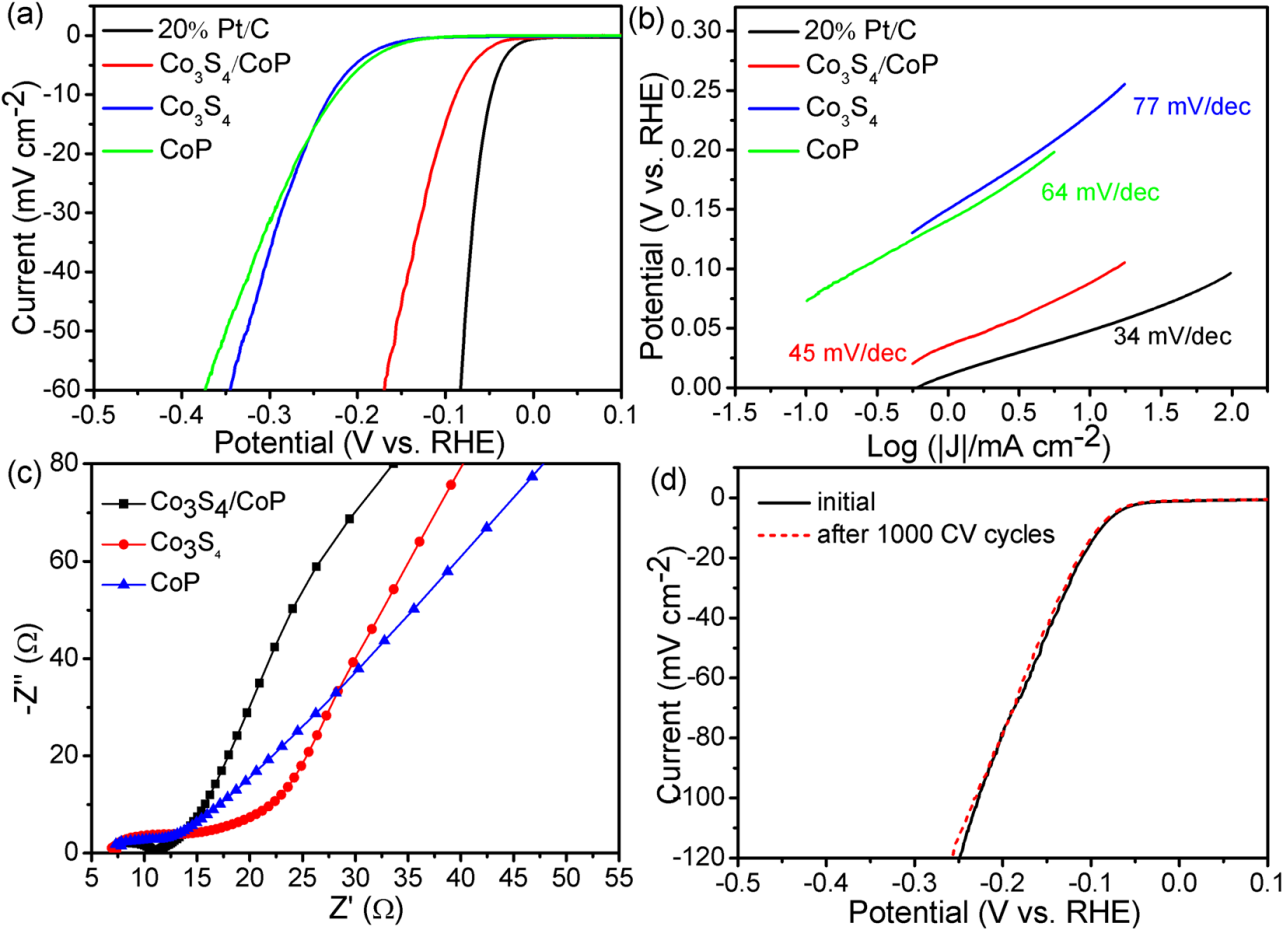
(**a**) LSV curves, (**b**) corresponding Tafel plots of the 20% Pt/C, Co_3_S_4_/CoP, Co_3_S_4_ and CoP catalysts derived from the polarization curves, (**c**) Nyquist plots and (**d**) stability test.

Two common indicators are adopted here to assess the electrocatalytic HER performance of the products. The onset overpotential is the potential at which current density begins to fall steeply due to proton reduction, and the Tafel slope is the increase in overpotential required to elicit a magnitude rise in current density^39,40^. Figure 6(a) shows the HER polarization curve of Co_3_S_4_/CoP catalyst obtained from linear sweep voltammetry (LSV) measurement with a sweep rate of 5 mV s^−1^, and pure Co_3_S_4_, CoP and 20% Pt-C catalysts were also tested for comparison with the same mass loading. Typically, the 20% Pt-C catalyst shows the highest HER performance with an ideal overpotential of zero. At the same time, also from the Fig. 6(a) we can see that the Co_3_S_4_/CoP hybrid catalyst exhibits a very low onset potential of about 38 mV versus RHE, which is far superior than that of Co_3_S_4_ (132 mV) and CoP (121 mV) based HER catalysts. This result demonstrates the structure compounded by Co_3_S_4_ and CoP can improve the HER performance effectively. In addition, due to the measurement in determining HER onset overpotential is not the same in different literatures, the overpotential at a current density of 10 mA cm^−2^ (η_10_) is employed as a benchmark to compare the HER performance with various electrocatalysts. As expected, the η_10_ of Co_3_S_4_/CoP is only 86 mV, which is much better than most of the reported cobalt-based HER electrocatalysts with similar catalyst loading in acid (Supplementary Table S1).

Generally, different values of Tafel slope can be obtained with the change of rate-determining step. By plotting overpotential against log current density (log |J|), the Tafel plots for assessing the HER kinetic are obtained, and the Tafel slope (b) can be calculated by fitting the linear portion according to the Tafel equation $\eta ={\rm{a}}+b\,\mathrm{log}\,j$, where b and j represent the Tafel slope and the current density, respectively. As shown in Fig. 6(b), the obtained Tafel slope are 34, 45, 64 and 77 mV dec^−1^ for the 20% Pt/C, Co_3_S_4_/CoP, CoP and Co_3_S_4_ catalysts, respectively, indicating that the HER kinetics of Co_3_S_4_/CoP hybrid is more favorable than pure CoP and Co_3_S_4_ catalysts. It is well known that there are three possible steps in acidic solutions for HER catalysts^41^, including Volmer, Heyrovsky and Tafel steps (equations (1–3)).

1 $${\rm{Volmer}}\,{\rm{reaction}}:{H}_{2}{O}^{+}+{{\rm{e}}}^{-}\to {H}_{ads}+{H}_{2}O$$

2 $${\rm{Hyrovsky}}\,{\rm{reaction}}:{H}_{2}O+{H}_{3}{O}^{+}+{{\rm{e}}}^{-}\to {H}_{2}+{H}_{2}O$$

3 $${\rm{Tafel}}\,{\rm{reaction}}:{H}_{{\rm{ads}}}+{H}_{ads}\to {H}_{2}$$

Generally, different rate determining steps in the HER process correspond to different values of Tafel slopes. When the discharge process is fast and followed by a slow electrochemical desorption or a slow atom combination reaction, the corresponding Tafel slope is 30 or 40 mV dec^−1^, respectively. Conversely, if the discharge process is difficult to implement, a large value of 120 mV dec^−1^ will be obtained. Based on the analysis, it can be sure that the HER process over Co_3_S_4_/CoP via the Volmer-Heyrovsky mechanism with the electrochemical desorption step as the rate-determining step^42^, and the existence of the bubbles hinders the adsorption of hydrogen atoms to some extent, which makes the Tafel slope obtained in experiment is slighter larger than theoretical value. The exchange current densities (j_0_) of samples were also obtained by extrapolating the Tafel plots at the equilibrium potential (i.e., at zero overpotential) and are listed in Table 1. The resulting j_0_ of the Co_3_S_4_/CoP catalyst is 0.15 mA cm^−2^, far above the value of pristine Co_3_S_4_ (10.3 μA cm^−2^) and CoP (7.8 μA cm^−2^), indicating that the electrons transferring on the interface of Co_3_S_4_/CoP only need a very low activation energy. Meanwhile, the HER kinetics at the electrode/electrolyte interface of Co_3_S_4_/CoP catalyst is further examined by the electrochemical impedance spectroscopy (EIS) in the frequency ranged from 10^6^ to 0.01 Hz. The Nyquist plots in Fig. 6(c) reveal that the Co_3_S_4_/CoP electrode exhibits a reduced charge-transfer resistance (R_ct_) of 4.1Ω as compared to that of the Co_3_S_4_ and CoP electrodes, demonstrating the highly efficient charge transport rate of Co_3_S_4_/CoP NRs. The smaller value of Rct of Co_3_S_4_/CoP electrocatalysts is attributed to their 1D porous structure, which can increase the contact of the active sites with the electrolyte, leading to a significant acceleration of the interfacial electrocatalytic reactions.

**Table 1:** Summary of the electrochemical parameters of Co_3_S_4_/CoP, Co_3_S_4_ and CoP.

Sample	C_dl_ (mF cm^−2^)	Onset overpotential (mV)	η_10_ (mV)	Tafel slope (mV decade^−1^)	j_0_ (μA cm^−2^)	R_ct_ (Ω)
Co_3_S_4_/CoP	21.04	38	86	45	150.4	4.1
Co_3_S_4_	1.31	132	231	77	10.3	9.7
CoP	0.32	121	223	64	7.8	6.0

Durability of the HER electrocatalysts is very important for their practical application in daily life. Therefore, the long-term stability of Co_3_S_4_/CoP hybrid catalyst is measured by subjecting it to CV cycling at a higher scanning rate, which is known as the accelerated degradation test, and to chronoamperometric or chronopotentiometric analyses^43^. It can be seen from Fig. 5(d) that there is almost no active loss to be observed by comparing the LSV curves of the Co_3_S_4_/CoP before and after 1000 CV cycles treated with a scan rate of 50 mV s^−1^, which indicates the good stability of catalyst based on Co_3_S_4_/CoP hybrid.

## Discussion

**Figure 7:**
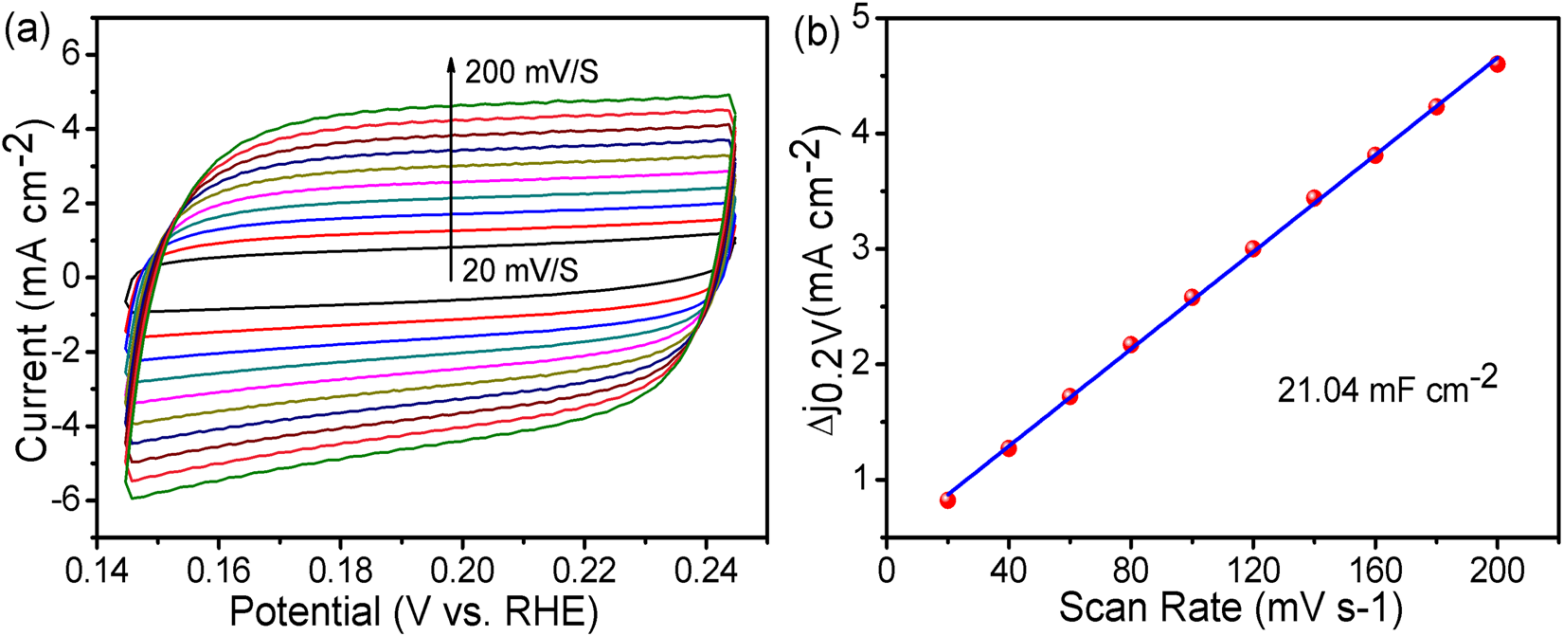
(**a**) CV curves of the Co_3_S_4_/CoP NRs with different scan rates (20–200 mV s^−1^) in the potential range 0.144–0.244 V vs RHE in 0.5 M H_2_SO_4_ solution at room temperature, (**b**) the corresponding linear relationship between current density variation and scan rate.

During the HER process, the appropriate ΔG_H*_ value of CoP shell and the attractive metallic nature of Co_3_S_4_ core provide a basic guarantee for the excellent HER performance of Co_3_S_4_/CoP hybrid catalyst^44^. However, the synergistic effect between the core and shell is still unclear. For this purpose, the effective electrochemical active surface area (ECSA), which is considered to be linearly proportional to double layer capacitance (C_dl_), is calculated^45–49^. Figure 7(a) shows the CV curves of the Co_3_S_4_/CoP catalyst at different sweep scan rates (20–200 mV s^−1^) in the potential range of 0.144–0.244 V vs RHE. All the observed curves display pseudo-capacitive characteristics without any obvious Faradaic peaks, and the area of the CV curves gradually expand with the increasing in scan rates. According to the CV curves and the calculation method reported^50^, the C_dl_ value of the Co_3_S_4_/CoP hybrid catalyst is calculated to be about 21.04 mF cm^−2^, which is much larger than that of pure Co_3_S_4_ (1.31 mF cm^−2^) and CoP (0.32 mF cm^−2^) catalysts (Supplementary Fig. S6). The increased C_dl_ value of Co_3_S_4_/CoP hybrid catalyst is mainly attributed to the synergistic effect between Co_3_S_4_ and CoP because, as the referential sample, pure CoP has the same surface morphology with Co_3_S_4_/CoP hybrid. Furthermore, the existence of pores enlarges the core-shell interface, and the small thickness of CoP shell could make the strong synergetic coupling effects revealed on the whole surface.

In summary, our results here raise a new strategy to synthesis nanocomposites effectively. The Co(CO_3_)_0.5_(OH)·0.11H_2_O precursor was firstly prepared by a hydrothermal method, and then the phosphidation with NaH_2_PO_2_ and sulphidation with S powder in Ar atmosphere were carried out simultaneously to obtain the Co_3_S_4_/CoP NRs. The results from the XRD, XPS and EDS mapping indicate that the obtained hybrid is of core-shell structure with CoP as the shell and Co_3_S_4_ as the core. Due to the synergetic effect arising from the core and shell, as well as the big electroactive surface area, the as-prepared Co_3_S_4_/CoP hybrid catalyst exhibits an excellent HER properties, including a low onset overpotential of 34 mV, a small Tafel slope of 45 mV dec^−1^, an exceptional low overpotential of 86 mV at a current density of 10 mA cm^−2^ with a mass loading of 0.28 mg cm^−2^, and permanently durability at least 1000 CV cycles durability in acidic media. Therefore, the current research suggests that the Co_3_S_4_/CoP hybrid NRs might be a promising replacement to the Pt-based catalysts for H_2_ production with little loss in electrical energy.

## Methods

### Materials synthesis

Cobalt (II) nitrate hexahydrate (Co(NO_3_)_2_·6H_2_O), Ammonium fluoride (NH_4_F), urea (CH_4_N_2_O), Sulfur powder (S), sodium monophosphate (NaH_2_PO_2_), and Sodium sulfide nonahydrate (Na_2_S·9H_2_O) were purchased from Shanghai Aladdin Bio-Chem Technology Co., Ltd. Sulfuric acid (H_2_SO_4_, 95–98%) and ethanol (99.9%) were acquired from Beijing Chemical Works. All chemicals were the analytical-grade reagents and used as received without any further purification.

### Preparation of cobalt precursor materials

Cobalt precursor materials were prepared using a method reported by Faber *et al*. with minor modifications^51^. Typically, 1.5 mmol Co(NO_3_)_2_·6H_2_O, 3 mmol NH_4_F and 7.5 mmol CH_4_N_2_O were dissolved in 50 mL distilled water and stirred adequately for 0.5 h at room temperature. Then, 35 mL of the mixture was transferred to a 50-mL PTFE-lined stainless steel autoclave and maintained at 120 °C for 6 h. After the autoclave cooled down naturally, the pink precipitates were collected, washed and dried for the later synthesis.

### Preparation of Co_3_S_4_/CoP, CoP and Co_3_S_4_ nanoturatures

#### Co_3_S_4_/CoP

Firstly, the as-prepared cobalt precursor and 0.5 g NaH_2_PO_2_ were put at two separate positions in an alumina boat, and 0.5 g S powder was placed in another. Then, the two boats were placed in the center of different heating zones of a double temperature zone tube furnace, respectively, and make sure their positions from upstream to downstream be S powder → NaH_2_PO_2_ → cobalt precursor (see Fig. 5(a)). After the furnace was purged of air under a steady flow of Ar carrier gas (99.999%) at 25 sccm, the upstream zone was heated to 500 °C at a ramping rate of 5 °C min^−1^ and the downstream was heated to 300 °C at a ramping rate of 3 °C min^−1^. Finally, the Co_3_S_4_/CoP NRs were obtained after the furnace cooled down to room temperature naturally.

#### Co_3_S_4_

Firstly, the cobalt precursor was thermally treated at 350 °C for 3 h in air to form Co_3_O_4_ nanorods. Then, the as-prepared Co_3_O_4_ were dispersed in the 30 mL aqueous solution of Na_2_S·9H_2_O, and transferred into an autoclave at 160 °C for 12 h. After cooling down to room temperature, the sample was filtered and dried at 60 °C. Finally, the Co_3_S_4_ nanostructure was obtained.

#### CoP

The CoP NRs were prepared by thermal annealing cobalt precursor (Co(CO_3_)_0.5_(OH)·0.11H_2_O) at 300 °C for 150 min in a phosphorus (1.12 g NaH_2_PO_2_) atmosphere.

#### Characterization

The synthesized materials were characterized by several techniques. The microstructures of the specimens were observed by X-ray diffraction (XRD, TD-3500, China) using Cu K_α_ radiation with the λ = 0.15418 nm in the 2θ scanning range from 10° to 70°. Scanning electron microscope (SEM, Carl Zeiss, Germany), transmission electron microscopy (TEM, Model JEOL–2010, Japan) and high resolution transmission electron microscopy (HRTEM) were carried out to observe the morphology of the specimens. Elemental distribution and chemical state were examined by energy-dispersive X-ray spectroscopy (EDS) mapping and X-ray photoelectron spectroscopy (XPS, UIVAC-Phi, Japan), respectively, and all binding energies were referenced to the C1s peak at 284.6 eV adventitious carbon to correct the shift caused by charge effect. The specific surface area was analyzed by Brunauer–Emmett–Teller (BET) method through nitrogen adsorption using the JW-004 instrument.

#### Electrochemical measurements

The electrochemical performance of the samples was carried out in a standard three-electrode electrochemical cell using an electrochemical workstation (CHI660D). A glass carbon electrode, Pt wire, and Ag/AgCl were used as working, counter, and reference electrodes, respectively. Typically, 4 mg of catalyst were dispersed in 1 mL of a mixture solution (V_water_:V_ethanol_ = 3:2). After adding 30 μL Nafion solution (0.5 wt%) and ultrasonication for 1 h, a homogeneous slurry was formed. Then, an aliquot of 5 μL was pipetted onto the glass carbon electrode and dried at room temperature (loading: 0.28 mg cm^−2^). All the potentials in our paper were corrected to the reversible hydrogen electrode (RHE) using the equation E (RHE) = E (Ag/AgCl) + 0.059 pH + 0.197 V.

## Electronic supplementary material

Supplementary information
