# Supplementary material for: An efficient Co3S4/CoP hybrid catalyst for electrocatalytic hydrogen evolution
Source: Sci Rep. 2017 Sep 19;7:11891. doi: 10.1038/s41598-017-12332-4 (PMC5605511; doi:10.1038/s41598-017-12332-4)
Supplement: Supplementary file 1 — Supplementary information [file 41598_2017_12332_MOESM1_ESM.pdf]

Supplementary Information for:

An efficient Co_3S_4/CoP hybrid catalyst for electrocatalytic hydrogen evolution

Tingting Wang[1], Liqian Wu[1], Xiaobing Xu[1, 2], Yuan Sun[1], Yuanqi Wang[1], Wei Zhong[1, *], Youwei Du[1]

[1]Collaborative Innovation Center of Advanced Microstructures, National Laboratory of Solid State Microstructures and Jiangsu Provincial Laboratory for NanoTechnology, Nanjing University, Nanjing, 210093, China.

[2]College of electronic Engineering, Nanjing Xiaozhuang University, Nanjing, 210017, China.

[*] Corresponding author. E-mail: *wzhong@nju.edu.cn*

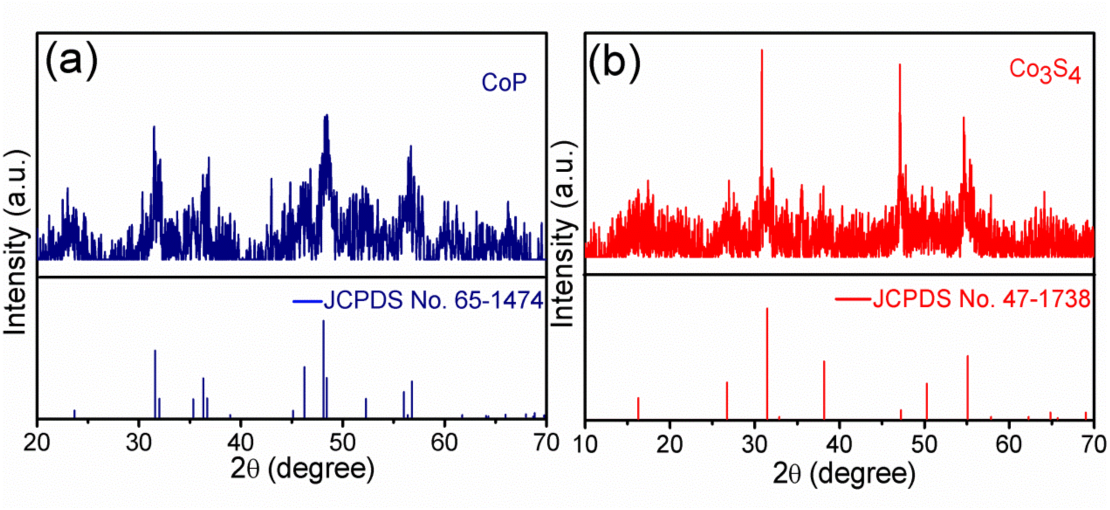

Figure S1. XRD patterns of the as-synthesized (a) CoP, and (b) Co_3S_4 catalysts.

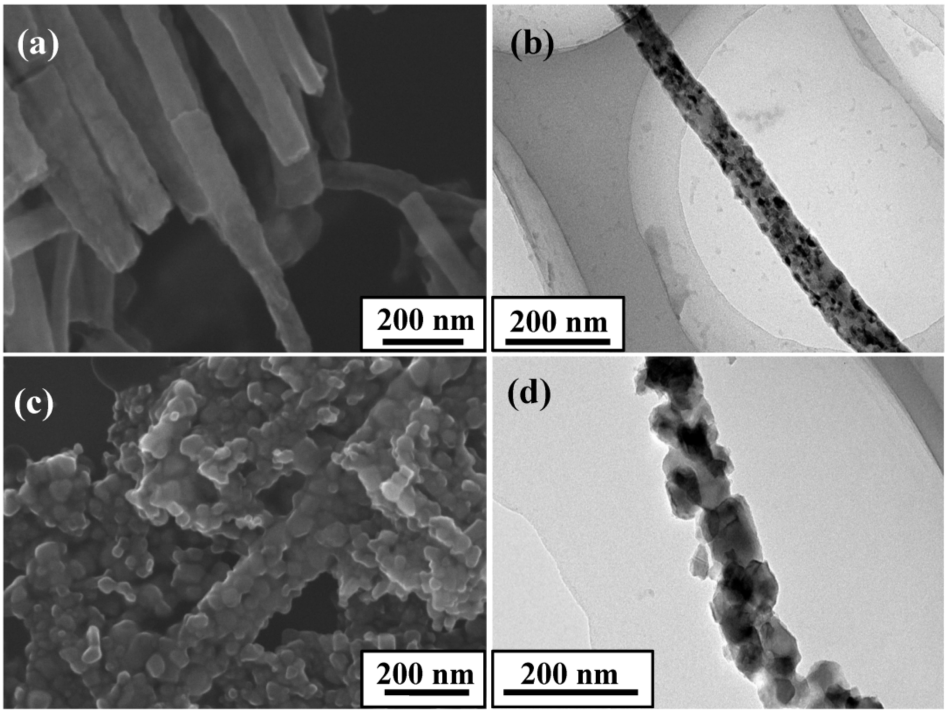

Figure S2. SEM and TEM images of CoP (a, b) and Co_3S_4 (c, d), respectively.

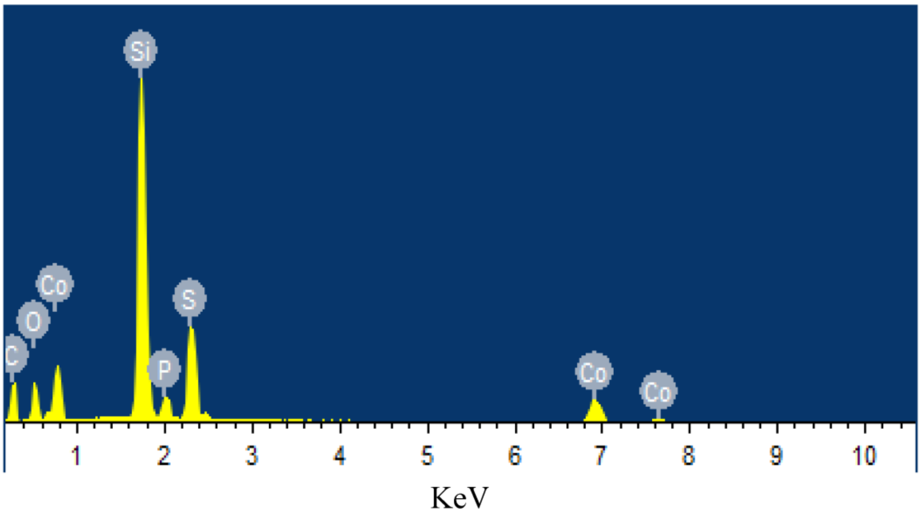

Figure S3. EDX spectrum of Co_3S_4/CoP NRs.

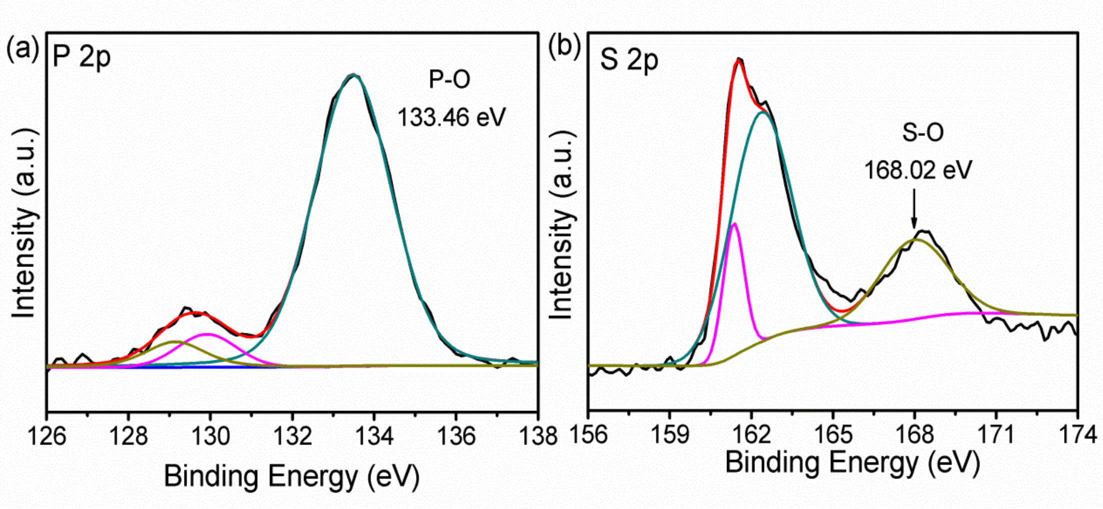

Figure S4. High-resolution XPS spectrum in the (a) P 2p region of CoP, and (b) S 2p region of Co_3S_4.

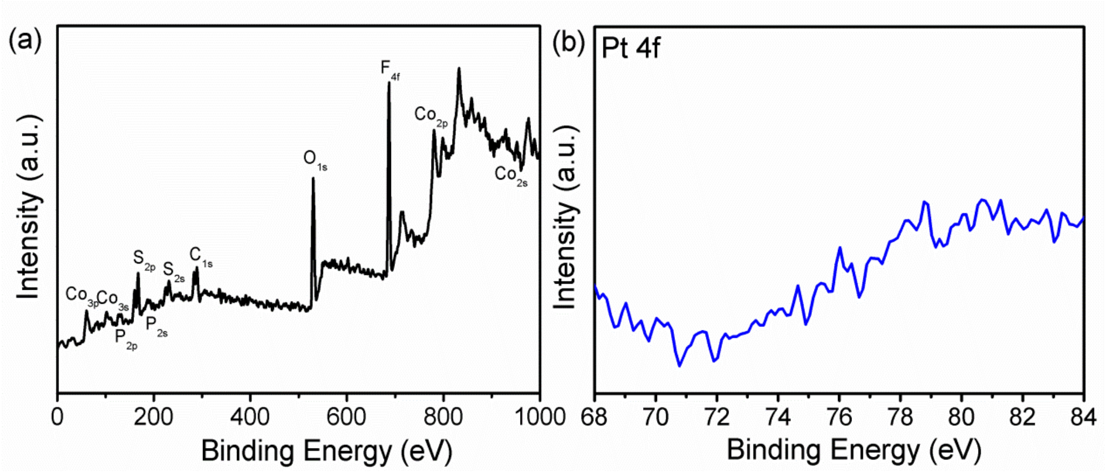

Figure S5. (a) XPS spectrum of Co_3S_4/CoP hybrid, and (b) high-resolution Pt 4f spectra after HER electrolysis, which excluded the possibility of Pt deposition during HER (F signal was from Nafion).

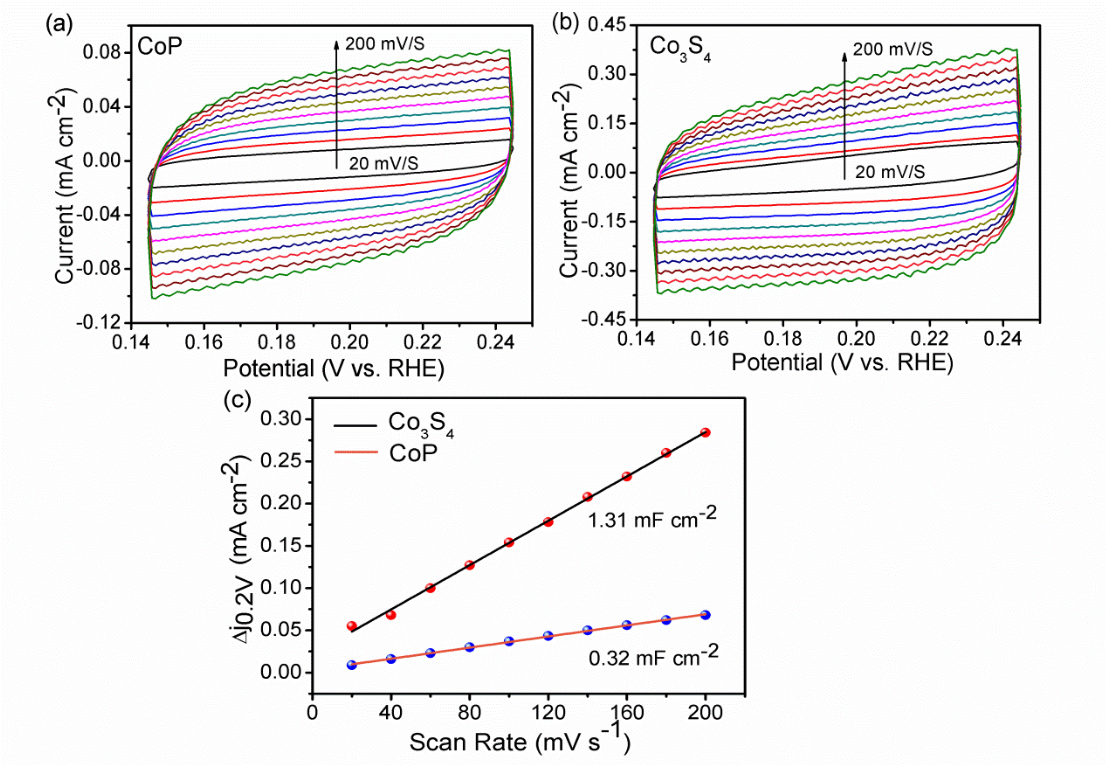

Figure S6. (a) CV curves of (a) CoP and (b) Co_3S_4 with different scan rates (20-200 mV s^{-1}) in the potential range 0.144-0.244 V vs RHE in 0.5 M H_2SO_4 solution, (b) the corresponding linear relationship between current density variation and scan rate.

Table S1. Comparison of selected cobalt-based HER electrocatalysts in 0.5 M H_2SO_4 solution.

Sample	η_{10} (mV)	Ref.	Sample	η_{10} (mV)	Ref.
HNDCM-Co/CoP	135	[1]	CoP/CNT	122	[6]
H-CoP/C	111	[2]	$Co_xS_y/WS_2/CC$	120	[7]
CoP/NPCF	135	[3]	Co_9S_8-30@MoS_x/CC	98	[8]
CoS_2/RGO	180	[4]	Co-N-P-CNFs	248	[9]
Co_3S_4 NCs	250	[5]	CoS_2	232	[10]
CoP microspheres	226	[6]	Co_3S_4/CoP	86	This work

References

[1] Wang H. et al. Nitrogen-doped nanoporous carbon membranes with Co/CoP Janus-Type nanocrystals as hydrogen evolution electrode in both acidic and alkaline environments. ACS Nano 11, 4358−4364 (2017).

[2] Bai Y.J. et al. Strengthened synergistic effect of metallic MxPy (M = Co, Ni, and Cu) and carbon layer via peapod-Like architecture for both hydrogen and oxygen evolution reactions. Small 13, 1603718−1603728 (2017).

[3] Lin Y., Pan Y., Zhang J. CoP nanorods decorated biomass derived N, P co-doped carbon flakes as an efficient hybrid catalyst for electrochemical hydrogen evolution. Electrochimi. Acta 232, 561–569 (2017).

[4] Yang Y.Y. et al. Porous CoS_2 nanostructures based on ZIF-9 supported on reduced graphene oxide: Favourable electrocatalysis for hydrogen evolution reaction. Int. J. Hydrogen Energy 34, 6665–6673 (2017).

[5] Pan Y., Liu Y.Q., Liu C.G. Phase- and morphology-controlled synthesis of cobalt sulfide nanocrystals and comparison of their catalytic activities for hydrogen evolution. Appl. Surf. Sci. 357, 1133–1140 (2015).

[6] Liu Q. et al. Carbon nanotubes decorated with CoP nanocrystals: a highly active non-noble-metal nanohybrid electrocatalyst for hydrogen evolution. Angew. Chem., Int. Ed. 53, 6710–6714 (2014).

[7] Shang X. et al. Novel Co_xS_y/WS_2 nanosheets supported on carbon cloth as efficient electrocatalyst for hydrogen evolution reaction. Int. J. Hydrogen Energy 42, 4165–4173 (2017).

[8] Zhou X.F. et al. Symmetrical synergy of hybrid Co_9S_8-MoS_x electrocatalysts for hydrogen evolution reaction. Nano Energy 32, 470–478 (2017).

[9] Wang Z. et al. Facile electrospinning preparation of phosphorus and nitrogen dual-doped cobalt-based carbon nanofibers as bifunctional electrocatalysts. J. Power Sources 311, 68–80 (2016).

[10] Kong D.S., Cha J.J., Wang H.T., Lee H.R., Cui Y. First-row transition metal dichalcogenide catalysts for hydrogen evolution reaction. Energy Environ. Sci. 6, 3553–3558 (2013).
